# Combining Microarray Technology and Molecular Epidemiology to Identify Genes Associated with Invasive Group B *Streptococcus*

**DOI:** 10.1155/2008/314762

**Published:** 2008-02-25

**Authors:** Lixin Zhang, Usha Reddi, Usha Srinivasan, Sheng Li, Stephanie M. Borchardt, Parvathy Pillai, Puja Mehta, Anne N. Styka, Joan DeBusscher, Carl F. Marrs, Betsy Foxman

**Affiliations:** ^1^Department of Epidemiology, University of Michigan School of Public Health, Ann Arbor, MI 48109, USA; ^2^Program in Bioinformatics, Eastern Michigan University, Ypsilanti, MI 48197, USA; ^3^Fargo VA Medical Center, Fargo, ND 58102, USA

## Abstract

Many bacterial species function as both commensals and pathogens; we used this dual nature to develop a high-throughput molecular epidemiological approach to identifying bacterial virulence genes. We applied our approach to Group B *Streptococcus* (GBS). Three representative commensal and one invasive GBS isolates were selected as tester strains from a population-based collection. We used microarray-based comparative genomic hybridization to identify open reading frames (ORFs) present in two sequenced invasive strains, but absent or divergent in tester strains. We screened 23 variable ORFs against 949 GBS isolates using a GBS Library on a Slide (LOS) microarray platform. Four ORFs occurred more frequently in invasive than commensal isolates, and one appeared more frequently in commensal isolates. Comparative hybridization using an oligonucleotide microarray, combined with epidemiologic screening using the LOS microarray platform, enabled rapid identification of bacterial genes potentially associated with pathogenicity.

## 1. INTRODUCTION

Group B *Streptococcus* (GBS), or *Streptococcus agalactiae*, a common bowel inhabitant, also frequently colonizes the vagina, urethra, and pharynx asymptomatically. However, GBS can cause a variety of invasive diseases that occur primarily in neonates, young infants, elderly persons, and pregnant women [1–3]. Host factors are clearly important as GBS disease occurs primarily in vulnerable populations. Nonetheless, bacterial virulence factors must also play a role: propensity to cause disease varies by serotype [4], and genetic sequence types that cross serotype, which have a high potential to cause invasive disease, have been identified [5].

Among the nine known GBS capsular serotypes, serotypes Ia, III, and V cause the majority of GBS disease in the United States [1, 6–8]. Studies of the population structure and the molecular epidemiology of GBS isolates suggest that GBS populations are clonal, but that some strains may be more virulent than others [9–13]. By pulsed-field gel electrophoresis (PFGE), disease-causing isolates have limited heterogeneity within a serotype (reviewed in Schuchat, 1998 [2]) while colonizing isolates are quite heterogeneous [14] within a particular serotype, suggesting that invasive isolates have distinctive features which enhance pathogenesis. Differences in virulence are likely related to the presence or absence of virulence genes [15]. Although advances have been made in the understanding of classic GBS traits, such as capsular polysaccharide, *β*-hemolysin, C5a peptidase, and immunogenic surface proteins [16–19], our understanding of the pathogenesis of GBS infections is limited: little is known about which bacterial genetic factors contribute to virulence or transmission of pathogenic strains.

The availability of complete and draft genome sequences of several GBS strains presents an opportunity to gain insight into the molecular basis of GBS virulence. Analysis of these genome sequences confirmed a high level of genetic heterogeneity among GBS strains, even of the same serotype [20]. The GBS genome contains a large number of genetic islands that often vary among strains and are likely to be the regions where virulence genes reside [21–23]. With thousands of genes identified in GBS genomes, the current challenge is to determine which are important in GBS pathogenesis and transmission. In a previous study of *E. coli*, we presented an approach of bacterial gene identification and evaluation that relied on epidemiologic information for selecting isolates for genomic subtraction and screening of epidemiologically defined collections for evaluation of the significance of genes identified through genomic subtraction [24]. In this report, we applied the same principle in a three-step strategy to the study of GBS. Further, we employed novel microarray platforms that enabled us to systematically identify candidate genes and evaluate their importance on a large scale.

Genome comparison between pathogenic and nonpathogenic strains within a species is a powerful strategy for identifying candidate genes important for virulence [25, 26]. Since none of the currently sequenced GBS genomes are from commensal isolates, we first selected a few representative colonizing GBS strains from a population-based sample for comparisons with sequenced pathogenic serotype III strain (NEM316) and serotype V strain (2306VR), two serotypes representing the most frequently encountered disease-causing isolates. We then identified sequence differences and their associated variable genes between selected colonizing and sequenced invasive GBS strains using comparative genomic hybridization with fine-tiling oligonucleotide microarrays. Lastly, a selected set of variable genes was screened against a large panel of colonizing and invasive strains using Library on a Slide microarray to evaluate their association with disease. Our main objective of this report is to use GBS as an example to demonstrate and evaluate this study approach.

## 2. MATERIALS AND METHODS

### 2.1. Bacterial strains and culture conditions

Disease-causing and commensal GBS isolates were selected from various collections obtained from previous epidemiologic studies. Collections included isolates from healthy male and nonpregnant female college students enrolled at the University of Michigan [27–30], isolates from symptomatic and asymptomatic pregnant women seen at a University of Michigan Medical Center clinic [31], and isolates collected from patients through the Wisconsin Invasive Bacterial Laboratory Surveillance System between 1998 and 2002 [32]. Additional isolates from newborns in Texas with early and late onset disease [8] as well as isolates from pregnant women with and without GBS disease were obtained from Dr. Carol J. Baker (Baylor College of Medicine, Houston, Texas, USA). These strains were broadly grouped into two categories: invasive isolates from patients with invasive diseases (*n* = 386) and colonizing isolates from subjects without any symptomatic diseases (*n* = 563). GBS strain NEM316 [21], 2603VR [23], and A909 [33] were used as reference strains. GBS isolates were cultured overnight in Todd-Hewitt broth (Oxoid) for DNA isolation.

### 2.2. PFGE and capsular typing

PFGE was performed as described previously [30]. Briefly, GBS DNA was digested with *Sma*I and electrophoresed for 18 hours (initial switch time 4 seconds; final switch time 16 seconds) with the CHEF III apparatus (Bio-Rad, Hercules, CA). Gels were strained for 4 h with Vistra Green (Amersham Biosciences, Piscataway, NJ, USA) at 4 C, and visualized with a Storm PhosphorImager (Amersham Biosciences, Piscataway, NJ, USA). PFGE patterns were analyzed using BioNumeric software (Applied Maths, Kortrijk, Belgium). A dendrogram was constructed using the unweighted pair group method with arithmetic means, Dice coefficient, optimization setting of 1.0%, and a position tolerance of 1.0%. GBS isolates were classified into capsular types Ia, Ib, and II–VIII using DNA dot blot hybridization, as previously described [34].

### 2.3. GBS oligonucleotide microarray construction

Two fine-tiling oligonucleotide microarrays were designed using the published DNA sequences for serotype III strain (NEM 316, GenBank accession no. AL732656) and serotype V strain (2603VR, GenBank accession no. AE009948). The NEM 316 array consisted of a total of 368,576 32-mer probes (184,288 pairs) tiling its 2.21 Mb genome every 12 bases for both strands. The 2603VR array consisted of a total 360,040 32-mer probes (180,020 pairs) tiling its 2.16 Mb genome every 12 bases for both strands. Arrays were designed and constructed through custom Array CGH service from NimbleGen (Madison, Wiss, USA) using its maskless array synthesis (MAS) technology. A denser tiling array with shorter oligonucleotides was usually used in NimbleGen's two-step comparative genome sequencing (CGS) [35].

### 2.4. Comparative genome hybridization and data acquisition

Comparative genomic hybridization and signal processing were performed by NimbleGen custom service (NimbleGen Systems Inc., Madison, Wiss, USA). Briefly, GBS DNA from four tester strains and two reference strains were broken down into separate pools of low molecular weight fragments, labeled independently with cyanine fluorescent dye and each was hybridized to one NEM316 and to one 2603VR whole-genome tiling array. Similar to the Affymetrix chips, the short oligo GBS arrays were produced by in-slide de novo syntheses. Like Affymetrix chip hybridization, one slide was used for hybridization per labeled sample. A total of 12 microarray hybridizations were performed. Genomic hybridization of NEM316 or 2603VR to its own array served as a signal reference for comparisons with tester strain hybridizations using the same array. Genome hybridization of one sequenced genome against the other sequenced genome array was used for validation purposes. The signal intensity ratios of tester DNA to each reference DNA were compared to identify probe sequences absent or different from the tester genome. The ratio was generated by normalizing the signal intensity (setting the median ratio to 1 and the standard deviation to 0.45), dividing the reference by the tester for each strand, and then averaging the two strands. Signal ratios were plotted as a function of their genomic positions and visualized using SignalMap software from NimbleGen. A custom algorithm was used to mark the potential variable probe sequence (absent or different in tester genome) based on comparison to a local threshold (a 1800 bp window). This analysis was also performed by NimbleGen (see Supplementary Material available at doi:10.1155/2007/14762 for the analytical algorithm).

### 2.5. Additional bioinformatic and data analysis methods

Genome-scale sequence comparisons between genomes of NEM316 and 2603VR were performed using GenomeComp [36]. All strain-specific genetic islands greater than 10 bp were identified using run parameters set to 0.01, −3, and 1 for expectation value (e), penalty for a mismatch, and reward for a match, respectively. To identify matches of probe sequences from one fine-tiling oligoarray in the other sequenced genome, a custom Bioperl program was used for local batch blast analyses. The stand alone BLAST program [37] for Unix operation system was used and the percent identity of the best hit for each probe within the query genome was determined. All other data analyses were performed using SAS v9.0 (SAS Institute, Inc Cary, NC, USA) and S plus v6.1 (Insightful Corporation, Seattle, Wash, USA).

### 2.6. GBS library on a slide microarray construction and hybridization

We recently developed a new application of microarray technology, called Library on a Slide (LOS), for bacterial comparative genomics studies [38]. LOS technology combines dot blot hybridization with the technology of microarrays resulting in glass slides with thousands of bacterial genomes arrayed. Thus libraries of entire genomes rather than the sequence of a single genome or set of genes are printed on the slides. Slides are used to screen large numbers of strains for the presence of specific genetic elements of interest. A GBS LOS microarray was created with genomic DNA from 949 GBS isolates sampled from a variety of GBS collections and various control strains. Genomic DNA was isolated using a high-throughput sonication-based method described previously [39]. DNA from GBS strains along with controls were arrayed in duplicate on Vivid Gene Array slides (Pall Life Sciences, Mich, USA) using a VersArray ChipWriter compact arrayer (Bio-Rad, Calif, USA). Selected GBS ORFs and four house keeping genes, (alcohol dehydrogenase (*adhP*), phenylalanyl tRNA synthetase (*pheS*), glutamine synthetase (*glnA*), and glucose kinase (*glcK*)), were PCR-amplified from either strain NEM316 or 2603VR. Purified PCR products were fluorescein-labeled using BioPrime DNA labeling kit (Invitrogen, Calif, USA). Each probe was hybridized with a different slide overnight at 68°C in PerfectHyb Plus hybridization buffer (Sigma, Mo, USA). After washing, fluorescein-labeled probes were detected using antiflourescein alkaline phosphatase (Roche, Switzerland) and alkaline phosphatase kit (TeleChem, Calif, USA). The intensity of each spot was normalized to the intensity of the quantification probe (a mixture of four housekeeping genes) to account for differences in DNA concentrations at different spots, and compared to the intensity of the positive control (sequence strain known to contain the gene probe) to determine presence/absence of the gene fragment in different bacterial strains using previously established methods [38, 40].

## 3. RESULTS AND DISCUSSION

### 3.1. Selection of tester strains for comparative genomic subtraction

Comparing pathogenic and nonpathogenic strains within a species can provide critical insights into bacterial pathogenesis. However, all sequenced GBS strains are from invasive diseases. We used molecular epidemiological comparisons to select representative commensal colonizing GBS isolates for the comparative genomic hybridization with the highest potential to identify potential pathogenesis-related genes in sequenced invasive genomes (strains NEM316 and 2603VR). We characterized the diversity of 882 colonizing isolates from a population-based longitudinal study of healthy male and nonpregnant female college students [28] using PFGE and serotyping. Clustering analysis by dendrogram was performed on these isolates along with a sample of 35 invasive isolates and sequenced pathogenic strains NEM316 and 2603VR. Based on this analysis, we selected three commensal isolates that are genetically distant from the two sequenced genomes but representative of isolates from relatively large strain clusters that are predominantly of commensal origin. Isolate 657–461 is a serotype V strain representing the largest clonal group within our commensal collection. Isolate G617-061 (serotype III) and G293-061 (serotype II) are from two additional clusters dominated by colonizing strains. In addition to these three commensal isolates, one invasive isolate, H-19, was chosen for the comparative genomic subtraction, because it represents the most common clonal type of the serogroup Ia strain in our analysis. Since serotype classification does not necessarily reflect genetic distance among strains [20], we did not select tester strains based solely on the differences in serotype. The small number of colonizing stains chosen here could not and was not intended to capture the diversity of commensal isolates. It was our first attempt at performing genome-wide comparisons between colonizing strains and sequenced genomes, so we could pick gene candidates from a list of several thousands for association study using LOS microarrays where large collections of population-based GBS isolates can be screened.

### 3.2. Oligonucleotide array validation

We used shared probe sequences within the two genome arrays to assess the reproducibility of the comparative genomic hybridization and used hybridization of one sequenced genome against the array of the other sequenced genome to evaluate the accuracy of the array in assessing sequence variation.

Between 184,288 probe pairs on the NEM316 array and 180,020 probe pairs on the 2603VR array, a total of 16,364 identical probe pairs (32/32 match) were identified. Hybridization results for this probe subset from two arrays for each tester genome were treated as replicas for accessing the reproducibility of CGH. Hybridization for each probe was classified as identical or variable in the tester genome when compared to the reference genome. The percent concordance for tester genomes G293-061, H1-19, G617-061, and G654-461 were 98.75%, 99.50%, 99.88%, and 98.62%, respectively. The reproducibility was very high even when the raw signal ratios were examined. The correlation coefficients were greater than 90% for duplicates.

To evaluate the accuracy of the array in assessing sequence variation, we compared results from in silicoanalysis with the classification results from an actual array hybridization swap between NEM316 and 2603VR genomes. Almost all perfectly matched probe sequences were correctly identified as identical by hybridization. Only 4 out of 133,520 and 5 out of 133,570 probe sequences were falsely identified as different with NEM316 and 2603VR arrays, respectively. However, 28,673 out of 50,768 (56%) and 25,435 out of 46,450 (55%) mismatched probe sequences were falsely identified as identical with NEM316 and 2603VR arrays, respectively. At the probe level, hybridization has a high sensitivity but low specificity for detecting conserved probe sequences. Nonetheless, the high-density nature of the tiling array still provides overall sequence variation information at genome and ORF levels. We visually displayed the CGH results by plotting hybridization signal ratios of the probes along their genomic positions and compared them with an in silico comparison of NEM316 and 2603VR. The majority of variable probes (i.e., probes with high reference versus tester signal ratios) are clustered primarily around strain-specific genetic islands identified by the in silico analysis. To convert probe-level variation to ORF sequence variation, we calculated the percentage of variable probes for each ORF (number of variable probes identified within an ORF divided by the total number of probes tiling the ORF). Using different percentage cutoff values in classifying variable ORFs (divergent or absent), the CGH-based data was compared with in silico analysis (Table 1). For the NEM 316 array, a 15% cutoff value gave a 2.9% false-negative rate (i.e., ORFs known to be present but classified by hybridization as absent or very divergent) and the best overall sensitivity and specificity (97% and 94%, resp.). For the 2603VR array, the 20% cutoff point gave the best overall sensitivity and specificity (97% and 91%, resp.). Thus, these two cutoff points were chosen to classify variable ORFs for the remaining CGH analyses.

Comparative genomic hybridization with fine-tiling 32-mer oligonucleotide microarrays did not identify all probe sequence variations in the two reference genomes but reliably identified variable ORFs using combined hybridization results of all probes within each ORF.

### 3.3. Distribution and mapping of variable probe sequences

**Figure 1:**
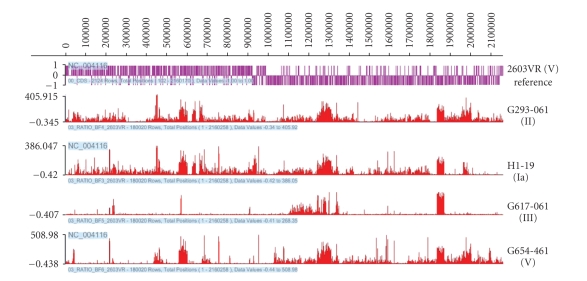
Locations of variable probes sequences identified within genomes of group B *Streptococcus* strains 2603VR in comparative genomic hybridization using each of the four tester strains of group B *Streptococcus*.

CGH using NEM316 and 2603VR genome arrays revealed that 3.4–15.4% of probe sequences were absent or divergent in the four tester strains (Table 2). This range of diversity is similar to the range of sequence differences (5% to 15%) recently observed in pairwise comparison of all eight available complete or draft GBS genome sequences [20]. The tiling arrays allowed a high-resolution view of genome variation among comparison strains. Figure 1 displays comparative hybridization results of tester strains against 2603VR by plotting the reference to tester signal ratios along their genomic positions. Although the four tester strains represent four different serotypes (Ia, II, III and V), the majority of absent or divergent probe sequences in these strains are mapped to the same set of regions on the NEM316 genome, a serotype III strain. To a lesser extent, the same set of regions on the 2603VR genome covers the majority of the probes that are absent or divergent across three of the four tester genomes. The strain G617-061, a serotype III strain, is very similar to the serotype V reference strain 2603VR genome, with only 3.4% probes identified as different compared to >13% of the other tester genomes. Comparison of all eight available complete or draft GBS genome sequences also demonstrated that serotype classification does not reflect the genetic diversity of the GBS [20]. One possible explanation for closely related strains exhibiting different capsules is genetic exchange of genes determining the capsular type by horizontal gene transfer.

### 3.4. NEM316 and 2603VR ORFs absent/divergent in tester genomes

Hybridization results from all the probes within each ORF were used to determine the presence or absence/divergence in the tester genome using the criteria established through analyzing control experiments (described above). Among the 2134 ORFs within the NEM316 genome, 484 (22.7%) were identified as variable ORFs because they were classified as absent/divergent in at least one tester genome. 269 (56%) of them were absent/divergent in four tester genomes, and 96, 84, and 35 were classified as absent/divergent in 1, 2, and 3 genomes, respectively. Of 2124 ORFs within the 2603VR genome, 530 (25%) were identified as variable ORFs. Among them, 81, 121, 162, and 166 were classified as absent/divergent in 4, 3, 2, and 1 tester genomes, respectively. Pairwise genome alignment of the two reference genomes identified strain-specific regions with a total length of 288 kb and 239 kb in NEM316 and 2603VR, respectively. Greater than 95% of the ORFs residing within these strain-specific regions were identified as variable ORFs in our CGH with four tester genomes, representing 64% (309/484) of NEM316 variable ORFs and 52% (275/530) of the 2603VR variable ORFs. About 80% of variable ORFs identified by CGH are located within fourteen putative pathogenicity islands previously identified in NEM316 [22].

**Figure 2:**
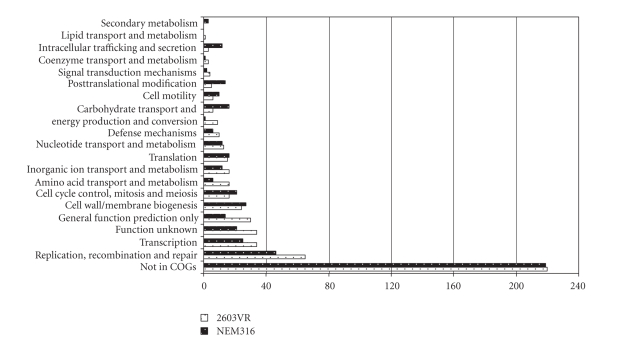
Number of variable open reading frames in group B *Streptococcus* strains NEM316 and 2603VR classified in each of the clusters of orthologous genes (COG) category plus those not classified in COG database.

To investigate which functional groups these variable ORFs belong to, we classified ORFs into clusters of orthologous genes (COGs) [41]. Figure 2 shows the number of variable ORFs in each COG category. Approximately half of the variable ORFs have not been classified into COGs and are of unknown function. The most common classifiable variable ORFs belong to the COG category of DNA replication, recombination, and repair. This is likely attributable to the presence of integrated phage or plasmids in the reference genomes. A large number of variable ORFs are predicted to be involved in transport, regulation, intermediate metabolism, and cell wall metabolism. These genes may be important in maintaining the pathogenic life style and transmission of the invasive GBS strains. Genes within these categories have also been identified through an in vivo study in which signature-tagged mutagenesis and a neonatal rat model were used to identify novel GBS genes implicated in virulence [42]. Relatively few variable ORFs were found to be involved in coenzyme transport and metabolism and lipid transport and metabolism.

### 3.5. ORFs absent/divergent in at least two tester genomes of commensal origin

ORFs consistently absent/divergent in commensal tester strains compared to invasive strains are likely virulence gene candidates. Six ORFs were absent/divergent in all three commensal tester strains, and conserved in the two invasive reference genomes and the invasive tester strain. We identified an additional 29 ORFs from the reference genomes that were absent/divergent in at least two out of the three commensal strains (Table 3). Fifteen of these 35 ORFs are predicted hypothetical to be proteins of unknown function. Several ORFs are predicted to be proteins involved in transport, metabolism, and other metabolic functions. Also included are two putative lipoproteins and two surface proteins. Gene gbs0850 is predicted to encode a fibrinogen binding protein and is identical to a previously identified *fbsB* gene [43, 44]. The best match for gbs0850 in 2603VR is ORF sag0832, which encodes a different variant of the fibrinogen binding protein. ORFs gbs2015 and gbs2016 are two adjacent genes with highly similar DNA sequences predicted to encode glycosyl transferases. These two genes are also found together in the 2603VR genome as sag2060 and sag2061.

While we identified a large number of variable ORFs, few were missing in all three or even two commensal tester strains. Because multiple genetic factors are involved in GBS virulence and multiple pathogenesis pathways involving different sets of virulence genes likely exist, we might expect different sets of virulence genes to be identified when different pairs of invasive and colonizing isolates are compared.

### 3.6. GBS library on a slide hybridization and differentially distributed variable ORFs

All variable ORFs are potential virulence gene candidates. Using a novel GBS LOS microarray platform with a large collection of GBS isolates, the importance of these variable ORFs can be efficiently evaluated. We present our initial evaluation of 23 of the 35 ORFs identified above using GBS LOS. We were not able to synthesize good probes with strong and specific signals in our initial attempts for the other 12 variable ORFs because of their small sizes or poor PCR amplifications. We therefore left them out of this initial LOS screening. The GBS LOS microarray contained genomic DNA from 949 GBS isolates printed in duplicate. Among them, 386 were isolates from patients with invasive diseases and 563 were commensal colonizing isolates. In addition, the LOS array contained DNA from various control strains. Table 3 lists the prevalence of 23 ORF in the overall GBS collection and their prevalence ratios in invasive strains compared to colonizing strains. sag2060, sag2061, sag0832, and gbs0474 appeared more frequently in invasive isolates than in colonizing commensal strains. By contrast, sag0814 was more frequently found among colonizing isolates than invasive isolates.

ORFs sag2060 and sag2061 are two putative glycosyl transferase genes. Glycosylation plays an important role in many biological processes in eukaryotes, and there is increasing evidence for a role of glycosylation in bacteria. Many surface expressed bacterial structures such as LPS, LOS, capsule, flagella, and pili in pathogenic bacteria are glycosylated [45–47]. Glycosylation can also be used by bacteria to inactivate antibiotics [48, 49]. Interestingly, the glycosyl transferase gene *lic2B* in *Haemophilus influenzae* was found more frequently among isolates causing otitis media than in throat isolates from children [50]. Future mechanistic studies should shed light on the roles of these GBS glycosyl transferases in pathogenesis.

The sag0832 is predicted to encode a fibrinogen binding protein. This gene has been suggested to be an important virulence gene for invasive GBS disease. In a murine model of sepsis, the wild-type strain was more virulent than the isogenic strain with this gene inactivated [44]. sag0832 was also shown to promote GBS invasion into epithelial cells in vitro [43]. In addition to the three ORFs encoding known proteins, two ORFs (sag0814 and gbs0474) encoding hypothetical proteins were differentially distributed between invasive and colonizing strains. Given the large numbers of ORFs of no known functions that exist in the sequenced genomes, this is not surprising. Some of these ORFs probably involve complicated traits that are difficult to observe in laboratory conditions and will be more readily identified using association studies. Interestingly, sag0814 is found more frequently in commensal strains than in invasive strains. It is possible that lack of this gene enhances virulence. During the process of commensal-to-pathogen evolution, bacteria not only acquire virulence genes but also shed genes via deletions [51]. Deletions of genes that facilitate a commensal lifestyle could provide an additional evolutionary pathway towards virulence. For example, deletion of lysine decarboxylase gene greatly enhanced the enterotoxin activity in *Shigella* in its evolution [51].

While five out of 23 variable ORFs were differentially distributed between invasive and commensal isolates, the associations of these ORFs with invasive isolates were neither exclusive nor strong. Such outcomes were not unexpected for several reasons. First, we anticipate some degree of random misclassification to decrease the observed associations because invasive strains can be also commensal, and noninvasive strains can become opportunistic pathogens. Second, similar to the existence of several distinct pathotypes within many bacterial pathogens, there may be many different pathotypes within GBS. One virulence gene may be strongly associated with strains within one specific GBS pathotype but the association is less pronounced when all invasive isolates are included in the analysis. Third, GBS pathogenesis is determined by not one but many virulence genes and any one gene may only contribute. We are in the process of screening these and more variable ORFs on an additional 2000 isolates in order to perform a more definitive analysis. In addition, incorporating the population structure of GBS could potentially enhance our analysis and help with interpretation.

Screening 23 ORFs against 949 isolates also revealed the striking genome content diversity of GBS. We assigned each isolate to a genotype based on the presence or absence of all 23 probed examined. A total of 503 genotypes were observed among 949 isolates. Using this classification, strains with the same PFGE patterns have different gene composition. Profiling GBS with a limited number of gene probes could therefore provide a highly discriminative typing method.

## 4. CONCLUSIONS

As increasing numbers of bacterial genomes are sequenced, postgenome research will focus on identifying virulence-related genes and the function of these genes. We used a three-step molecular epidemiological approach employing two novel microarray platforms, fine-tiling oligonucleotide microarrays, and Library on a Slide to identify bacterial virulence genes potentially contributing to GBS disease. Among hundreds of variable ORFs identified by CGH, 35 were absent/divergent in two of out three commensal test strains but present in two invasive reference genomes and a tester invasive strain. We screened 23 of these ORFs against 949 GBS isolates, and found 5 ORFs that were differentially distributed between invasive and commensal isolates. We demonstrated that this approach can rapidly identify and evaluate bacterial genes potentially associated with pathogenicity.

In our approach, we adopted microarray-based CGH instead of the traditional genomic subtraction method to identify genetic differences between paired commensal and invasive GBS strains. The traditional genomic subtraction approach can sample only a fraction of strain-specific genes. The high-density tiling oligonucleotide array-based CGH allowed us to identify the complete array of DNA sequences unique to an invasive compared to a commensal GBS isolate. A denser and shorter oligonucleotide array design followed by a verification oligonucleotide array can be used for identifying even single nucleotide polymorphisms in the genome [35]. However, CGH-based genome comparisons depend on the availability of sequenced genomes, and moreover, strain-specific genes identified are confined to the sequenced genome. We were not able to detect and identify potential virulence genes that are likely to exist in other unsequenced pathogenic GBS strains. Given the pan-genome nature of the GBS species where the pool of variable genes are extremely large [20], future genome comparisons of any two strains are likely to rely on a cheap and fast direct sequencing approach such as pyrosequencing [52]. This new approach will eliminate the limitations of using CGH.

Once a set of candidate genes are identified by a genomic comparison, an even more critical step is to evaluate the role that these genes play in disease pathogenesis. This can be done by large-scale association studies, bioinformatic prediction, or biological functional analyses. Bioinformatics prediction requires databases with solid structural and functional information on biological molecules. Functional approaches are often limited to the presence of a characterizable virulence phenotype. Comparing gene frequencies among bacterial isolates collected from different sources, for example, disease-causing and commensal isolates, using statistical association, can provide insight into the relative importance of a gene sequence in pathogenesis and transmission. The number of isolates and diversity of the collections are important in determining the significance of observations made and in ensuring that there is sufficient power to detect associations. Large population-based samples are required to minimize the identification of spurious associations that often arise with small sample comparisons. Including commensal isolates (i.e., nondisease-causing strains) for study is an integral part of this approach to understand bacterial pathogenesis. The LOS microarray platform is a robust system, adaptable to a wide variety of bacterial pathogens, for detecting the presence or absence of a candidate gene in thousands of isolates efficiently, thus providing a truly high throughput system to evaluate genes in the postgenome era.

## Supplementary Material

NimbleGen uses custom software to identify potential different probe sequences in the tester strains. Briefly, the average reference/tester ratio of both forward and reverse sequence probes was calculated to identify a difference in a probe sequence between tester and reference strains. A threshold for a difference callâ was established based on the signals within 1800 bp of the current position. The tester probe sequence is classified to be different from reference if its average signal ratio is greater than the threshold. This process is iterated for all base pairs in the genome to calculate local thresholds for all probe sequences.

## Figures and Tables

**Table 1:** Sensitivity (probability open reading frame is detected, given it is truly present) and specificity assessments (probability open reading frame is not detected, given it is not present) of different cutoff values in classifying variable open reading frames using fine-tiling oligonucleotide genome arrays created from the genomic sequence of group B *Streptococcus* strains NEM316 and 2603VR.

Reference genome	Percentage cutoff point	Sensitivity	Specificity
NEM316	20%	0.98	0.91
	15%	0.97	0.94
	10%	0.96	0.96

2603VR	20%	0.97	0.91
	15%	0.95	0.92
	10%	0.89	0.96

**Table 2:** Number (percentage) of variable probe sequences among four tester group B *Streptococcus* genomes using sequenced strains as a reference revealed by comparative genomic hybridization.

Reference/sequenced genomes of invasive isolates	H1-19 (Ia) (invasive)	G293-061 (II) (commensal)	G617-061 (III) (commensal)	G654-461 (V) (commensal)
2603VR	327 (15.4%)	320 (15.1%)	72 (3.4%)	278 (13.1%)
NEM316	277 (13.0%)	305 (14.3%)	305 (14.3%)	271 (12.7%)

**Table 3:** Open reading frames (ORF) present in invasive strains but absent at least in two out of three commensal tester group B *Streptococcus* strains by comparative genomic hybridization and their presence among 949 GBS isolates and their prevalence ratio between invasive (*n* = 386) and colonizing (*n* = 563) isolates.

ORF	Probe-positive strains (%)	Prevalence ratio (95% CI)^(a)^	Predicted protein
sag0004	524 (55%)	1.1 (0.96–1.22)	hypothetical protein
sag0005	706 (74%)	1.0 (0.93–1.09)	hypothetical protein
sag0027	941 (99%)	1.0 (0.98–1.00)	phosphoribosylaminoimidazole synthetase
sag0175	692 (73%)	1.0 (0.93–1.09)	hypothetical protein
sag0206	590 (62%)	0.9 (0.82–1.01)	lipoprotein, putative
sag0253	(b)	(b)	acetyltransferase, GNAT family
sag0414	927 (98%)	1.0 (0.97–1.01)	phosphorylase, Pnp/Udp family, putative
sag0426	—	—	cupin family protein
sag0427	517 (54%)	1.0 (0.93–1.17)	transcriptional regulator, MerR family
sag0700	925 (97%)	1.0 (0.97–1.02)	2-dehydro-3-deoxyphosphogluconate aldolase/4-hydroxy-2-oxoglutarate aldolase
sag0814	117 (12%)	0.6 (0.39–0.83)	hypothetical protein
sag0815	364 (38%)	0.9 (0.76–1.06)	transcriptional regulator, Cro/CI family-related protein
sag0832	371 (39%)	1.5 (1.29–1.77)	fibrinogen binding protein
sag1130	367 (39%)	1.1 (0.90–1.25)	hypothetical protein
sag1140	(b)	(b)	hypothetical protein
sag1207	(b)	(b)	hypothetical protein
sag1781	(b)	(b)	primase-related protein
sag1968	87 (9%)	1.1 (0.71–1.61)	hypothetical protein
sag1969	907 (96%)	1.0 (0.97–1.03)	ribosomal protein L11 methyltransferase
sag1974	(b)	(b)	MutT/nudix family protein
sag1975	(b)	(b)	hypothetical protein
sag1976	290 (31%)	0.9 (0.73–1.08)	hypothetical protein
sag1994	289 (30%)	1.0 (0.82–1.22)	hypothetical protein
sag1999	(b)	(b)	hypothetical protein
sag2021	395 (42%)	1.2 (1.00–1.36)	cell wall surface anchor family protein
sag2026	224 (24%)	1.1 (0.88–1.40)	membrane protein, putative
sag2027	(b)	(b)	ABC transporter, ATP-binding protein
sag2028	(b)	(b)	hypothetical protein
sag2045	364 (38%)	1.1 (0.90–1.25)	DNA topology modulation protein FlaR, putative
sag2057	(b)	(b)	leucyl-tRNA synthetase
sag2060	427 (45%)	1.3 (1.13–1.50)	glycosyl transferase, family 8
sag2061	437 (46%)	1.2 (1.07–1.41)	glycosyl transferase, family 8
sag2088	(b)	(b)	hypothetical protein
sag2147	687 (72%)	1.0 (0.9–1.06)	lipoprotein, putative
gbs0474^(c)^	270 (28%)	1.5 (1.21–1.80)	hypothetical protein

^(a)^Prevalence with confidence interval not overlapping 1 are considered statistical significant.

^(b)^These ORFs were not screened using LOS mostly because of small sizes.

^(c)^Exact corresponding gene in strain 2603VR was not found by blast search while it was classified as present in 2603 VR by CGH.
