# Supplementary material for: Combining Microarray Technology and Molecular Epidemiology to Identify Genes Associated with Invasive Group B Streptococcus
Source: Interdiscip Perspect Infect Dis. 2008 Feb 25;2008:314762. doi: 10.1155/2008/314762 (PMC2648626; doi:10.1155/2008/314762)
Supplement: Supplementary file 1 — NimbleGen uses custom software to identify potential different probe sequences in the tester strains. Briefly, the average reference/tester ratio of both forward and reverse sequence probes was calculated to identify a difference in a probe sequence between tester and reference strains. A threshold for a difference callâ was established based on the signals within 1800 bp of the current position. The tester probe sequence is classified to be different from reference if its average signal ratio is greater than the threshold. This process is iterated for all base pairs in the genome to calculate local thresholds for all probe sequences. [file 314762.f1.pdf]

SUPPLEMENTAL DATA

Algorithm to determine comparative genomic hybridization probe sequence is different between tester and reference genome:

The custom software used to select CGH probes as different performs the following tests on the reference (Ref) and tester (Test) genome data:

1) Load Reference and tester probe intensities. Create a list of forward and reverse data. Ensure that for each position in the RefFwd, there is one in RefRev, TestFwd and TestRev.

2) Calculate Ratio for Ref/Test for Fwd and Rev. Calculate Average of these 2 numbers.

3) Get 50% Global median of RatioFwd, RatioRev, RatioAve as MedianGlobalFwd, MedianGlobalRev, MedianGlobalAve.

4) Calculate Global Threshold as described below and StdDev under threshold of RatioFwd, RatioRev, RatioAve as ThreshGlobalFwd, ThreshGlobalRev, ThreshGlobalAve.

5) Iterate through list of Data to calculate local thresholds for difference Calls. The threshold is calculated using the data that is within 1800 bp of the current position as described below. In those cases where all data is above the cutoff, the global Threshold is used. If the RatioAve is greater than ThresholdAve then the probe sequence is identified as different.

Threshold calculation:

1) The 80th percentile of the list is calculated as baseline1

2) The Std Dev is calculated as StdDev1

3) Any value above (baseline1 + StdDevMultiplier * StdDev1) is removed from the list.

4) The 80th percentile of the list is calculated as baseline2

5) The StdDev is calculated as StdDev2

6) The resulting Threshold is (baseline2 + StdDevMultiplier * StdDev2).

The StdDevMultiplier = 3.5
